# Sympathetic Ophthalmitis, and Certain Forms of Chronic Iritis

**Published:** 1860-10

**Authors:** E. L. Holmes

**Affiliations:** Professor of Surgery in Rush Medical College; Chicago


ARTICLE 2.

### SYMPATHETIC OPHTHALMITIS, AND CERTAIN FORMS OF CHRONIC IRITIS.

BY E. L. HOLMES, M. D., CHICAGO.

(Continued from page 513.)

### II—Chronic Iritis.

Inflammation of the iris must always be regarded as one of the most grave diseases to which the eye is liable. It terminates, when not arrested by suitable treatment, invariably, almost, with great diminution or complete loss of sight. Fortunately, the early stages of this affection, *uncomplicated*, yield satisfactorily, in a majority of instances, to appropriate remedies, and the eye escapes without permanent injury.

Still, we often meet with patients suffering from chronic iritis and the consequent impairment of vision. For, with the most careful attention on the part of the physician and patient, the inflammation will occasionally become chronic and obstinately resist the influence of every remedy; patients will not unfrequently apply for advice who have been unskillfully treated by ignorant practitioners, or who have wholly neglected medical treatment till a cure had become entirely hopeless; patients will often be imprudent and from necessity or carelessness, expose themselves to the exciting causes of relapse. It is well known that the iris, during convalescence from an attack of inflammation, is for a long time liable to relapse from slight causes, even when it has, to all appearances, assumed its normal aspect. Two of the severest cases of secondary iritis I ever saw were caused by acknowledged imprudence on the part of the patients themselves. The eye in each case, had almost entirely recovered. Exposure to cold winds and bright sun-light reproduced the most violent symptoms, which continued for a long time, and finally left the eye with vision permanently injured. From these, and perhaps other causes, every surgeon with extensive ophthalmic practice, meets with a large number of cases of chronic iritis. These cases, when apparently relieved, are exceedingly liable to relapse, each attack leaving the eye in a worse condition that before.

Von. Graefe, of Berlin, after a careful investigation of the subject for several years, with an opportunity of analyzing a large number of cases, made the important discovery *that adhesions of organized lymph between the lense and iris, was the predisposing cause of relapse in chronic iritis.* However difficult may be the explanation, there is no doubt of the fact, since the observations of v. Graefe have been numerous and have been verified by many ophthalmologists in England and on the continent of Europe. With regard to this disease it may be stated generally, that "Iritis, which has healed without adhesions between the lense and iris, is comparatively seldom subject to relapses: Iritis, with only minute elastic adhesions, will occasionally be in danger of secondary attacks: but when the disease is followed with extensive adhesions, which withstand the influence of atropine in dilating the pupil, it is almost uniformly liable to relapse. There is scarcely an exception to this rule, when the whole pupilary edge of the iris is attached to the lense, or when the pupil is much contracted and closed with lymph."

In these cases the relapse occurs from the slightest cause, or even from no apparent cause. The patient experiences, especially at night, dull pain, to a greater or less degree, in the eye or in the temple and brow. There is usually photophobia, with tenderness on pressure over the region of the ciliary processes. The sclerotia, near the circumference of the cornea, assumes a light red or pinkish color. The iris, as also the aqueous humor, becomes discolored. These symptoms increase in severity, or possibly continue for a long time without much change, annoying the patient, and even when only one eye is affected, rendering the other eye, (by sympathy,) almost entirely useless, till relief is obtained, which is, in most instances, merely temporary.

In these cases, as in the cases of foreign bodies in the globe, the tissues which are principally implicated are the iris and choroid. Disorganization of these and the other structures of the eye, often progresses till the eye becomes atrophied and completely amaurotic, when all inflammatory action may possibly cease. In cases of penetrating wounds of the eye, with persisting inflammation and obstinate chronic iritis, with total loss of vision, the removal of the affected eye, as we have stated, is probably the best course of treatment for the preservation of the other eye.

But in chronic iritis, (irido-choroiditis,) *with extensive adhesions of lymph*, where there is still a certain amount of vision, or perception of light, the treatment which v. Graefe adopted, and which he employed in a large number of cases before publishing his results, consists in removing about one-sixth of the iris, as in forming an artificial pupil.

Why this operation should afford so great relief in so many cases, is somewhat difficult to explain. We will not tax the patience of our readers with a sketch of mere theories, but refer those interested in the subject to the paper of v. Graefe, in particular "Archiv f. Ophthalmologie," vol. ii, part 2; to the London Ophthalmic Hospital Reports, vol. i, pages 57 and 216, and also to Desmarres' work on Diseases of the Eye, vol. iii, page 371.

At present it is our object simply to state the fact that in very many cases after the operation, the conjestion diminishes, the eye becomes less painful, the iris and aqueous humor assumes a more natural aspect, the organized lymph disappears, and the vision is restored, to a greater or less extent, according to the previous condition of the eye.

The operation, although simple, requires considerable care and delicacy of manipulation, and should only be performed after the more acute symptoms have somewhat subsided.

It is usually advisable to administer chloroform, to insure perfect control over the movements of the eye. A small incision, a line or two in length, is made into the anterior chamber, by passing the point of a lance-like knife through the sclerotic a half line behind the cornea. With care there is no danger of entering the posterior chamber, since the plane of the iris lies behind the union of the sclerotic and cornea. Through the opening thus made a fine pair of curved forceps is passed as far as the pupilary edge of the iris, for the purpose of seizing and withdrawing a portion of this membrane, which should be excised close to the cornea, by means of a pair of scissors. When the whole cornea is transparent the lower and inner portion of the iris should be selected for removal. In cases of opacity, the artificial pupil should, of course, be made behind the most transparent portion of the cornea.

After the operation, the lids should be kept closed for three or four days, by means of strips of delicate plaster, and the eye surrounded with wet compresses.

There is very little danger of subsequent inflammation. In three cases of my own, and in many cases which I have seen under the care of others, I have never observed any ill effects from the excision of a portion of the iris, as above described.

It is of great importance to the patient that the operation be performed before the disease has too far advanced. Whenever there is atrophy of the globe or whenever the posterior chamber is separated from the anterior by occlusion of the pupil from organized lymph, forcing the iris anteriorly towards the cornea, an operation seldom affords relief.

In the last number of the *Journal* we called the attention of its readers to a few facts regarding the *prevention* of sympathetic ophthalmitis. In reference to the *curative* treatment of this disease, when it is once established, we have only to add that there is scarcely a hope of benefit except from the excision of a portion of the iris. In many instances the congestion and neuralgic pain can thus be reduced and loss of vision prevented. It is almost needless to add that this operation should be preceded by the removal of the eye-ball primarily affected.

We earnestly urge upon our readers a careful consideration of the subjects of this paper. Although there are, comparatively, few patients suffering from the diseases of which we have spoken, it must be remembered that every physician *may* meet with them, and in more than one instance, by skillful treatment avert so great a calamity as blindness.

